# Supplementary material for: Polarization- and Chaos-Game-Based Fingerprinting of Molecular Targets of Listeria Monocytogenes Vaccine and Fully Virulent Strains
Source: Curr Issues Mol Biol. 2023 Dec 13;45(12):10056–78. doi: 10.3390/cimb45120628 (PMC10742786; doi:10.3390/cimb45120628)
Supplement: Supplementary file 1 [file cimb-45-00628-s001.zip › cimb-2711976-supplementary.pdf]

current issues in molecular biology

MDPI

Supplementary Materials to "Polarization- and chaos-game-based fingerprinting of molecular targets of Listeria monocytogenes vaccine and fully-virulent strains"

Dmitry A. Zimnyakov[1,2], Marina V. Alonova[1], Maxim S. Lavrukhin[2], Anna M. Lyapina[2] and Valentina A. Feodorova[2,3,*]

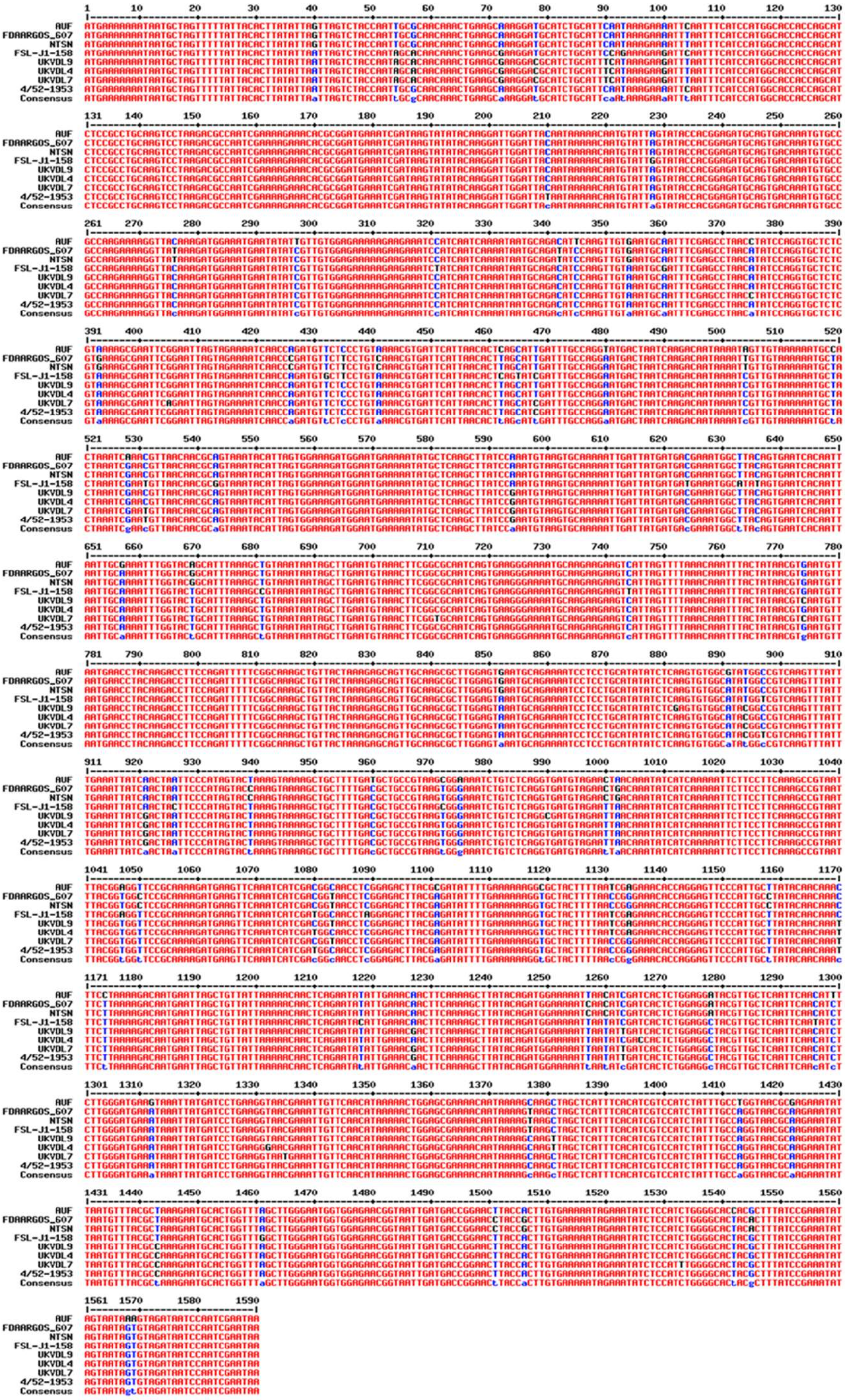

(a)

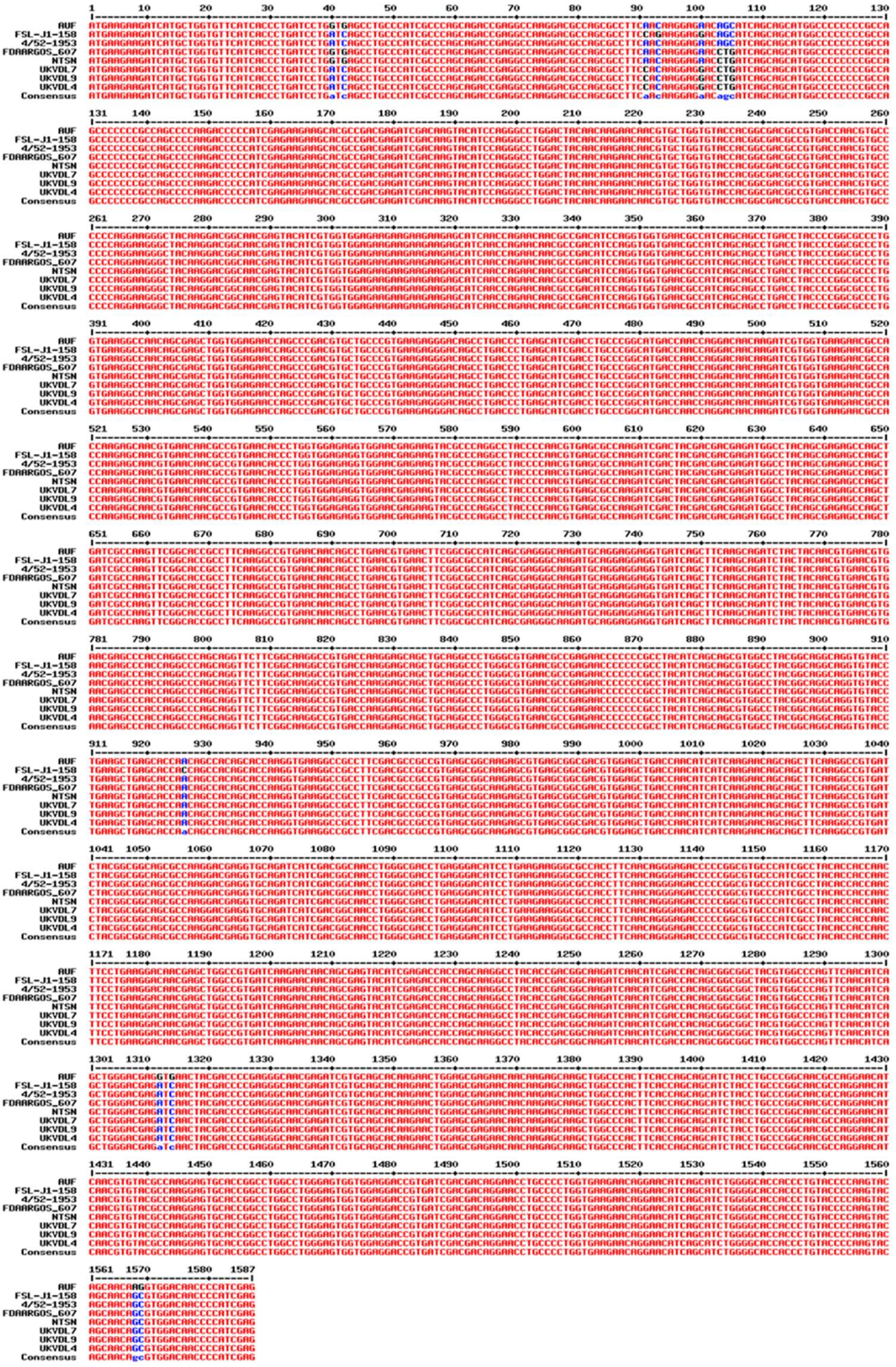

(b)

Figure S1. Multiple sequence alignments of the original (a) *hly* gene nucleotide sequences of the *L. monocytogenes* vaccine strain AUF and seven wild type *L. monocytogenes* strains of zoonotic origin and artificially generated sequences (b) obtained using Multalin online service (http://multalin.toulouse.inra.fr/multalin/). The SNPs are colored in blue.

Table S1. The homology level between the original AUF reference hly gene sequences and those derived from wild type *L. monocytogenes* strains of zoonotic origin (Fig. S1, a)

##	*L. monocytogenes* strain	Number of SNPs/mutations comparing to the AUF	Identity value between AUF and wild type L. monocyto-genes strains sequences*
1	AUF	0	100% (1590/1590)
2	FSL-J1-158	56	96.5% (1534/1590)
3	FDAARGOS_607	44	97.2% (1546/1590)
4	NTSN	44	97.2% (1546/1590)
5	UKVDL9	51	96.8% (1539/1590)
6	UKVDL4	48	97.0% (1542/1590)
7	4/52-1953	45	97.2% (1545/1590)
8	UKVDL7	55	96.5% (1535/1590)

*** Using the EMBOSS Needle on-line tool (https://www.ebi.ac.uk/Tools/psa/emboss_needle/**

Table S2. The homology level between the artificially generated sequences (Fig. S1, b)

##	Original sequence	Number of SNPs/mutations comparing to the AUF	Identity value between AUF and wild type *L. monocytogenes* strains sequences*
1	AUF	0	100% (1587/1587)
2	FSL-J1-158	10	99.4% (1577/1587)
3	FDAAR-GOS_607	7	99.6% (1580/1587)
4	NTSN	7	99.6% (1580/1587)
5	UKVDL9	11	99.3% (1576/1587)
6	UKVDL4	11	99.3% (1576/1587)
7	4/52-1953	6	99.6% (1581/1587)
8	UKVDL7	11	99.3% (1576/1587)

*** Using the EMBOSS Needle on-line tool (https://www.ebi.ac.uk/Tools/psa/emboss_needle/**

Table S3. The pairwise differences in nucleotides for the original hly *L. monocytogenes* gene sequences (Fig. S1, a; Table S1)

	1	2	3	4	5	6	7	8
1	0	56	44	44	51	48	45	55
2	56	0	54	54	41	36	37	43
3	44	54	0	0	43	42	37	45
4	44	54	0	0	43	42	37	45
5	51	41	43	43	0	9	20	12
6	48	36	42	42	9	0	19	17
7	45	37	37	37	20	19	0	18
8	55	43	45	45	12	17	18	0

Table S4. The pairwise differences in nucleotides for artificially generated sequences (Fig. S1, b; Table S2)

	1	2	3	4	5	6	7	8
1	0	10	7	7	11	11	6	11
2	10	0	9	9	5	5	4	5
3	7	9	0	0	4	4	5	4
4	7	9	0	0	4	4	5	4
5	11	5	4	4	0	0	5	0
6	11	5	4	4	0	0	5	0
7	6	4	5	5	5	5	0	5
8	11	5	4	4	0	0	5	0
